# Characterization of the complete chloroplast genome of *Microcycas calocoma* (Zamiaceae), an Endangered monotypic cycad species from Cuba

**DOI:** 10.1080/23802359.2019.1679683

**Published:** 2019-10-23

**Authors:** Jian Liu, Anders J. Lindstrom, Xun Gong

**Affiliations:** a Key Laboratory for Plant Diversity and Biogeography of East Asia, Kunming Institute of Botany, Chinese Academy of Sciences, Kunming, Yunnan, China;; b Key Laboratory of Economic Plants and Biotechnology, Kunming Institute of Botany, Chinese Academy of Sciences, Kunming, Yunnan, China;; c Nong Nooch Tropical Botanical Garden, Chon Buri, Sattahip, Thailand

**Keywords:** Cycad, Cuba, Critical Endangered, *Microcycas calocoma*, chloroplast genome

## Abstract

*Microcycas calocoma* is the monotypic species from the critical endangered and endemic cycad genus *Microcycas* in Cuba, an important taxon to study the evolution of extant gymnosperms. Here we report the complete chloroplast sequences of *M. calocoma* and characterize the genome structure of this species. The genome size of *M. calocoma* is 165,667 bp in length which contains 135 genes, including 88 protein-coding genes, eight ribosomal RNA genes and 39 transfer RNA genes. The GC content of this genome is 39.6%.

Phylogenomic study shows *M. calocoma* is mostly closely related to the cycad genus *Zamia*, which corresponds to previous studies based on multiple nuclear genes and combined plastid and nuclear evidence. The plastome information of *M. calocoma* offered by this study can contribute to further comparative chloroplast genome in cycads/gymnosperms as well as conservation genetic studies of *M. calocoma*.

*Microcycas calocoma* (Miq.) A.DC was first described by F.A.W. Miquel as the name *Zamia calocoma* (Miquel 1852) and formally named as *M. calocoma* by De Candolle under the monotypic genus *Microcycas* (De Candolle 1868). Based on the assessment of IUCN red list, this species is considered to be Critically Endangered, with a geographical range restricted to a total of five known localities in Western Cuba in the province of Pinar del Rio (Bösenberg 2010).

On account of the particular phylogenetic position of cycads, the cycad plastomes can be informative to trace the lineage history of extant gymnosperms and to study the plastid structure evolution in seed plants. However, *Microcycas* is the only genus among extant cycads whose chloroplast genome information is unavailable. Here, we assembled and characterized the complete chloroplast (cp) genome of *M. calocoma* in order to provide information for further conservation genetics within *M. calocoma* as well as comparative plastid genomic studies in seed plants.

Fresh leaf tissues were collected in Nong Nooch Tropical Botanical Garden, Thailand from cultivated plants (Acc. 456, BK) introduced from wild-collected plants in Consolacion del Sur of Cuba (22°33′11″N, 83°32′16″ W). Total genomic DNA was extracted by modified CTAB method (Doyle 1991). A total of 2 G raw data from Illumina Hiseq Platform (Novogene, Beijing, China) were screened and assembled into contigs by *Get_Organelle* pipeline (Jin et al. 2018) with published *Zamia furfuracea* (JX416857) as reference. The resulted contigs were reordered and further trimmed according to the *Z. furfuracea* reference to obtain the complete chloroplast genome. We applied PGA (Qu et al. 2019) to annotate the *Microcycas* plastid genome by using *Z. furfuracea* as reference. The annotations were double-checked in Geneious v11.0.3 (Kearse et al. 2012) and deposited in NCBI under the accession MN428318. To infer the intergenic relationship of all extant cycads, we used MAFFT v7 (Katoh and Standley 2017) to align an 11-plastome sequence dataset including all cycad genera and *Ginkgo*. ML tree inference was implemented in IQTREE 1.6.12 (Nguyen et al. 2015) with 1000 bootstrap replicates.

Based on the assembling results, the complete chloroplast genome of *M. calocoma* has typical quadripartite structure with a length of 165,667 bp, which contains 135 genes (88 protein-coding genes, eight rRNA genes and 39 tRNA genes). Among them, there are 10 genes (*rps16*, *atpF*, *rpoC1*, *petB*, *petD*, *rpl16*, *rps12*, *rpl2*, *ndhB*, *ndhA*) which have a single intron, while two genes (*clpP* and *ycf3*) were found to have two introns. This genome has two inverted repeat (IR) sequences, consistent with all other reported cycad plastomes (Wu and Chaw 2015). The overall GC content of *M. calocoma* genome is 39.6%, with the number 42% in IR, 38.8% and 37.1% in LSC (large single copy) and SSC (small single copy) regions, respectively. Phylogenomic analysis revealed a well-resolved tree within cycads (Figure 1). *Microcycas* was strongly supported as a sister with *Zamia* which agrees with the previous phylogenetic inferences of cycads based on both multiple single-copy nuclear genes (Salas-Leiva et al. 2013) and concatenated chloroplast and nuclear datasets (Liu et al. 2018).

**Figure 1.**
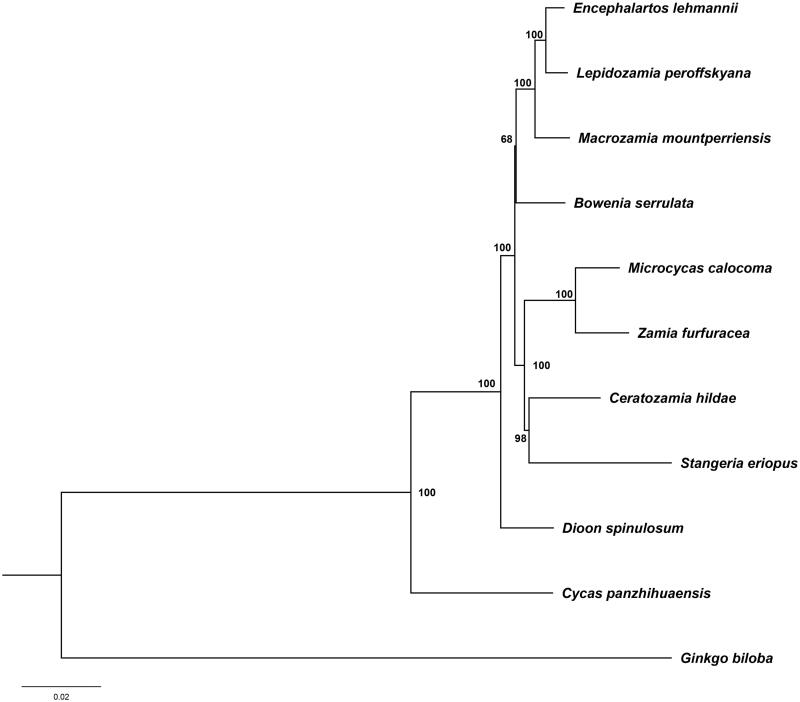
Intergeneric phylogenetic relationships across cycads based on the maximum-likelihood (ML) analysis of whole sequence evidence. Numbers on nodes represent for the bootstrap values based on 1000 replicates from IQTREE. Accession numbers: *Bowenia serrulata* NC_026036; *Ceratozamia hildae* NC_026037; *Cycas panzhihuaensis* KX_138991; *Dioon spinulosum* NC_027512; *Encephalartos lehmannii* LC_049336; *Lepidozamia peroffskyana* NC_027513; *Macrozamia mountperriensis* NC_027511; *Stangeria eriopus* LC_049067; *Zamia furfuracea* JX_416857; *Ginkgo biloba* JN_867585.

The complete chloroplast genome of *M. calocoma* in this study can contribute to further conservation of genetic studies of this species and also provide essential data for plastid comparative genomic studies across cycads/gymnosperms.
